# Combined Effects of Simulated Microgravity and Radiation Exposure on Osteoclast Cell Fusion

**DOI:** 10.3390/ijms18112443

**Published:** 2017-11-18

**Authors:** Srinivasan Shanmugarajan, Ye Zhang, Maria Moreno-Villanueva, Ryan Clanton, Larry H. Rohde, Govindarajan T. Ramesh, Jean D. Sibonga, Honglu Wu

**Affiliations:** 1 NASA Johnson Space Center, Houston, TX 77058, USA; srinimag@gmail.com (S.S.); maria.moreno-villanueva@uni-konstanz.de (M.M.-V.); jean.sibonga-1@nasa.gov (J.D.S.); 2 Department of Biological and Environmental Sciences, University of Houston Clear Lake, Houston, TX 77058, USA; Rohde@uhcl.edu; 3 NASA Kennedy Space Center, Cape Canaveral, FL 32899, USA; ye.zhang-1@nasa.gov; 4 Department of Biology, University of Konstanz, 78457 Konstanz, Germany; 5 Department of Nuclear Engineering, Texas A & M University, College Station, TX 77843, USA; rc1025@tamu.edu; 6 Department of Biology, Norfolk State University, Norfolk, VA 23504, USA; gtramesh@nsu.edu

**Keywords:** microgravity, radiation, osteoclast

## Abstract

The loss of bone mass and alteration in bone physiology during space flight are one of the major health risks for astronauts. Although the lack of weight bearing in microgravity is considered a risk factor for bone loss and possible osteoporosis, organisms living in space are also exposed to cosmic radiation and other environmental stress factors. As such, it is still unclear as to whether and by how much radiation exposure contributes to bone loss during space travel, and whether the effects of microgravity and radiation exposure are additive or synergistic. Bone is continuously renewed through the resorption of old bone by osteoclast cells and the formation of new bone by osteoblast cells. In this study, we investigated the combined effects of microgravity and radiation by evaluating the maturation of a hematopoietic cell line to mature osteoclasts. RAW 264.7 monocyte/macrophage cells were cultured in rotating wall vessels that simulate microgravity on the ground. Cells under static 1g or simulated microgravity were exposed to γ rays of varying doses, and then cultured in receptor activator of nuclear factor-κB ligand (RANKL) for the formation of osteoclast giant multinucleated cells (GMCs) and for gene expression analysis. Results of the study showed that radiation alone at doses as low as 0.1 Gy may stimulate osteoclast cell fusion as assessed by GMCs and the expression of signature genes such as tartrate resistant acid phosphatase (*Trap*) and dendritic cell-specific transmembrane protein (*Dcstamp*). However, osteoclast cell fusion decreased for doses greater than 0.5 Gy. In comparison to radiation exposure, simulated microgravity induced higher levels of cell fusion, and the effects of these two environmental factors appeared additive. Interestingly, the microgravity effect on osteoclast stimulatory transmembrane protein (*Ocstamp*) and *Dcstamp* expressions was significantly higher than the radiation effect, suggesting that radiation may not increase the synthesis of adhesion molecules as much as microgravity.

## 1. Introduction

All living organisms on Earth undergo physiological changes in response to the space environment, microgravity in particular. In humans, spaceflight has resulted in complications such as cardiovascular deconditioning, reduced immune functions, and unbalanced bone and mineral turnover [1,2]. Of the health risks associated with space travel, alterations in the skeletal mass may be a risk factor for secondary osteoporosis [3,4]. Bone remodeling is a dynamic process with a balanced removal of old bone by osteoclasts followed with new bone formation by osteoblasts. In terms of osteoblasts, glucocorticoids are known inhibitors of osteoblast cell growth, and microgravity has been shown to systemically increase cortisol levels in osteoblast cultures in space [5]. In space, microgravity has been shown to promote osteoclast activities in vivo [6]. Enhanced differentiation of bone-resorbing osteoclasts has also been reported using in vitro cell models and simulated microgravity on the ground [7]. Such differentiations are associated with tumor necrosis factor-related apoptosis inducing ligand (TRAIL) expressions. Despite numerous in vivo and in vitro studies attempting to explain the bone loss phenomenon in space, a mechanism of these cellular changes has remained elusive [8]. Furthermore, the responses to microgravity in specific bone regions need to be investigated [9].

In addition to bone loss due to microgravity, cosmic radiation is another challenging factor in the spaceflight environment, and radiation-induced bone loss in astronauts might be an additional risk factor for osteoporosis. Space radiation consists of mostly high-energy protons and other heavier charged particles of high linear energy transfer (LET) [10]. Ionizing radiation has been shown to contribute significantly to bone homeostasis. For example, in women treated for a variety of pelvic tumors, ionizing radiation increased the 5-year incidence of hip fracture by 65% [11]. In mice, exposure to 2 Gy γ rays, protons, carbon, or iron radiation species caused a 30–40% loss of their trabecular bone volume fractions [12]. Furthermore, exposures of mouse bones to X-rays resulted in an increase in the osteoclast number and activity [11]. Irradiation of a single-limb in a murine model induced local and paradoxically systemic bone loss [13]. Even though these are clinically relevant doses, recent publications reported that spaceflight-relevant radiation doses also promote low bone turnover and osteoclast activity [14].

Whether exposures to microgravity and space radiation simultaneously produce additive or synergistic consequences has been investigated with a number of biological endpoints such as DNA damage response [15]. With regard to bone loss, low doses of high-LET radiation, in conjunction with partial-weight bearing, appeared to promote the induction of bone loss with an increase in sclerostin-positive osteocytes and wnt-signaling [16]. In a mouse model looking at the tibia bone surface, radiation caused a 46% increase in osteoclast number, hindlimb-unloading caused a 47% increase in osteoclast number, and the combination of radiation and hind-limb unloading caused a 64% increase in osteoclast number [17]. A possible mechanism of synergy between microgravity and radiation is the fact that hindlimb unloading and radiation both cause increases in oxidative stress [17]. Although animal studies in both microgravity environment and with radiation exposure have given selective evidence about changes in osteoclast functions, the fusion mechanism for the differentiation to osteoclasts, as influenced by microgravity and/or radiation exposure in the space environment is still poorly understood.

Osteoclasts are multinucleated giant cells formed by a group of mononuclear osteoclast precursor cells fusing in a differentiation process [18]. These monocyte-macrophage cells fuse to form multinucleated osteoclasts, which act upon bone surfaces for effective bone resorption. Cell fusion is a complex progress that involves several genes. The dendritic cell-specific transmembrane protein (DCSTAMP) and osteoclast stimulatory transmembrane protein (OCSTAMP) have been discovered as fusogens for osteoclast differentiation [19,20,21]. Along with fusion, OCSTAMP appears to be necessary for optimal bone resorption [20]. The connective tissue growth factor (CCN2/CTGF) protein is a matricellular protein involved in intercellular signaling and plays an important role in skeletal development [22]. CCN2/CTGF expression during osteoclastogenesis promotes osteoclast formation via induction of and interaction with DCSTAMP [23]. Osteoclasts precursors differentiate into mature multinucleated osteoclasts after stimulation with macrophage colony stimulating factor (M-CSF) and receptor activator of nuclear factor-κB ligand (RANKL). RANKL is produced by bone marrow stromal cells. Immune cells can bind to receptor activator of nuclear factor-κB (RANK) present on the surface of osteoclast precursors and trigger cascade of downstream signaling for osteoclast function [24]. RANKL is also responsible for the formation of heterogeneous population of DCSTAMP^lo^ and DCSTAMP^hi^, which allow for the fusion process to occur between multinucleated cells [19].

Our present study made use of the RAW 264.7 murine cell line, which is a pure macrophage/monocytic and has a pre-OC population. The cells can develop into highly bone-resorptive osteoclasts upon RANKL stimulation [25,26]. Microgravity on the ground was simulated by culturing the cells in rotating wall vessels (RWV) [27]. This microgravity-modeled system has been used for several cell types and the studies performed have revealed strikingly similar results to those obtained during spaceflight [28]. A Cs-137 γ source was used to deliver radiation to the cells.

This study was aimed specifically at investigating the combined effects of microgravity and radiation exposure on osteoclast fusion.

## 2. Results

### 2.1. Cell Growth

**Figure 1:**
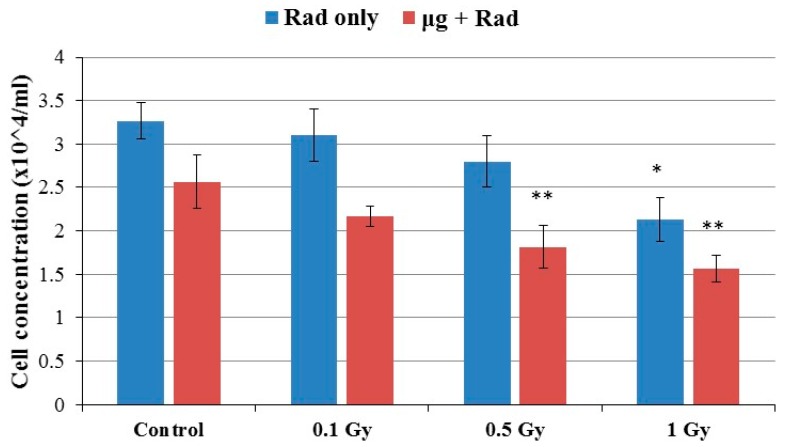
Concentration of RAW264.7 cells after exposure to different radiation doses. The number of cells decreased with increasing radiation doses. Cells previously cultured under simulated microgravity grew significantly slower (two-way ANOVA: *p* < 0.0001). Error bars mean standard deviation (SD) from three replicates. One-way ANOVA: *p* = 0.0038 for radiation only and *p* = 0.0026 for radiation + microgravity. Stars mean Dunnett’s multiple comparison test: * *p* < 0.05 and ** *p* < 0.01, compared to the corresponding control.

The number of cells/mL was determined after allowing 5-day cell culture with an initial concentration of 3 × 10^3^ cells/mL. The cell concentration decreased significantly (one-way ANOVA: *p* = 0.0091 for radiation only and *p* = 0.0027 for microgravity and radiation conditions) as radiation dose increased. Furthermore, the assessed cell concentration was significantly (two way ANOVA: *p* < 0.0001) lower (0.7–1.0 × 10^4^ cells/mL) when cells were previously incubated in simulated microgravity, indicating that microgravity itself negatively affected cell growth (Figure 1). Although cells grew slower after 1 Gy, the cell concentration reached 2.1 × 10^4^ cells/mL, which was 65.6% of the non-radiated cells.

### 2.2. Simulated Microgravity Increases Osteoclast Fusion

**Figure 2:**
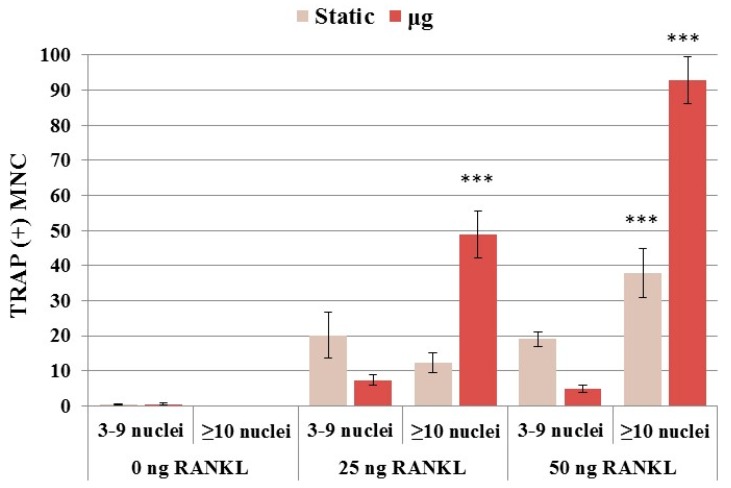
RANKL-dependent multinucleated cells formation under static and simulate microgravity. multinucleated cells (MNCs) with ≥10 nuclei significantly increases in microgravity compared to static condition (two-way ANOVA: *p* < 0.0001). The number of MNCs containing ≥10 nuclei increased significantly with increasing RANKL concentration in both static (one-way ANOVA: *p* = 0.0002) and microgravity (one-way ANOVA: *p* = 0.0001) conditions. Stars mean statistical significance compared to the corresponding control using Dunnett´s multiple comparison test (*** *p* < 0.005).

RAW 264.7 monocyte/macrophage cells were cultured in RWV or in static condition, and then treated with or without differentiation factor RANKL (25 or 50 ng/mL) for 5 days for the osteoclast fusion index. As shown in Figure 2, RANKL is needed for the multinucleated cell (MNC) formation. Although RANKL of 25 and 50 ng/mL induced similar numbers of MNCs containing 3–9 nuclei, the higher concentration apparently promoted more GMCs (Figure 2). For both concentrations, cells in the RWV culture condition demonstrated a significant three-fold increase (two-way ANOVA: *p* < 0.0001) in GMCs having 10 or more nuclei when compared to static cells. Furthermore, daily observation of the cells indicated that cell fusion in simulated microgravity started from 24 h of the culture, whereas in the static cells multinucleation began on Day 3. Our data support previous reports that microgravity stimulates increased osteoclast differentiation [26,29]. For studies of the combined radiation and microgravity effects, a RANKL concentration of 50 ng/mL was used.

### 2.3. Radiation Exposure Increases Osteoclast Fusion

**Figure 3:**
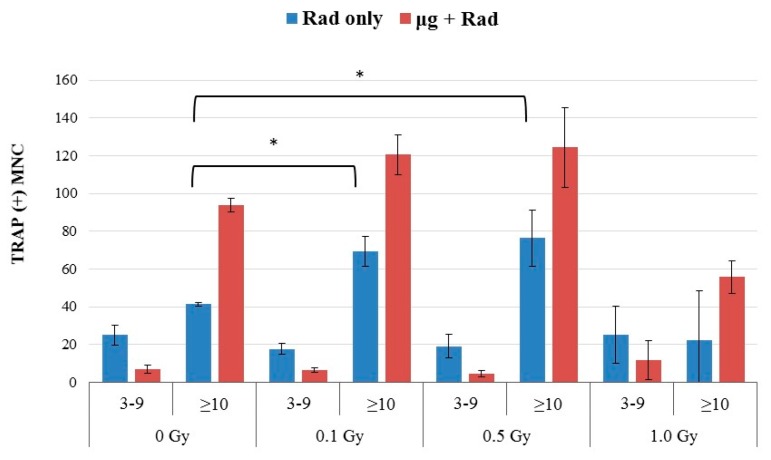
Induction of osteoclast fusion after radiation exposure static (blue bars) and under microgravity (red bars). 0.1 Gy and 0.5 Gy radiation significantly (* *p* < 0.05 *t*-test compared to 0 Gy) stimulated osteoclast fusion, but not 1 Gy. The number of multinucleated cells containing ≥10 nuclei increased significantly in radiation + simulated microgravity conditions compared with radiation alone (two-way ANOVA: *p* = 0.0001). Error bars mean SD from three independent experiments.

Without simulated microgravity culture, radiation alone increased osteoclast differentiation. As shown in Figure 3, γ rays of 0.1, 0.5, and 1.0 Gy had little impact on the number of MNCs containing 3–9 nuclei. However, the number of GMCs increased significantly even for doses as low as 0.1 Gy, The GMC number appeared to peak at doses of around 0.5 Gy and decreased at doses of 1 Gy.

### 2.4. Effects of Combined Radiation Exposure and Simulated Microgravity

In RAW 264.7 cells cultured in simulated microgravity, radiation exposure had little impact on the number of MNCs containing 3–9 nuclei, similar to static culture controls (Figure 3). The number of GMCs, however, were higher in RWV cells in comparison to the static culture controls, for both the irradiated cells and non-irradiated controls. The GMC number after combined radiation and microgravity exposures also peaked at doses of around 0.1–0.5 Gy.

### 2.5. Osteoclast Fusion Genes Up-Regulated in Microgravity

**Figure 4:**
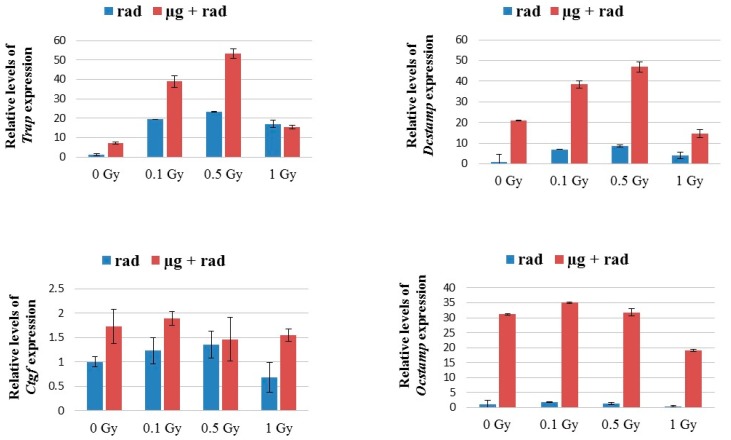
Expression of *Trap*, *Ocstamp*, *Dcstamp*, and *Ctgf* genes in response to different doses of gamma irradiation under the static (blue bars) and simulated microgravity (red bars) conditions. The increase in gene expression peaked at doses of 0.1–0.5 Gy. For all genes, except for *Ctgf*, expression was significantly higher in radiation + microgravity compared to radiation alone (two-way ANOVA of triplicates per each condition and dose; *p* = 0.0001).

RNAs collected 5 days after osteoclast culture were analyzed for *Trap*, *Dcstamp*, *Ocstamp*, and CGTF fusion gene expressions. Simulated microgravity alone increased the *Trap* gene expression in comparison to the static controls (Figure 4), in agreement with previous reports [26]. Radiation alone also activated *Trap* expressions, even at doses of 0.1 Gy, which is consistent with the GMC formation in the present study. The expression level of *Trap* was higher in cells after combined exposures to radiation and simulated microgravity in comparison to the cells exposed to radiation alone. *Trap* expressions peaked around 0.1–0.5 Gy for both gravity culture conditions. 

Similarly, simulated microgravity alone demonstrated increased *Dcstamp* and *Ocstamp* fusion gene expressions (Figure 4). Radiation alone also upregulated the expression of both genes in a dose dependent manner similar to *Trap* expressions. However, expressions after combined exposure to microgravity and radiation showed that the contribution from microgravity was significantly greater than radiation, particularly for *Ocstamp*. Simulated microgravity and/or radiation exposure had little impact on CCN2/CTGF expression levels (Figure 4).

## 3. Discussion

Microgravity and cosmic radiation are two of the most recognized environmental stress factors experienced during space travel. Microgravity is known to cause bone loss, but whether and how space radiation contributes to the potentially deleterious effects is still unclear. Loss of bone volume has been reported for low-LET radiation at doses of 1 Gy or above [30,31], and for high-LET radiation at doses below 1 Gy [16,32]. X-rays [33] and γ rays [34] have also been reported to increase osteoclast numbers in animals.

In this study, we investigated the formation of multinucleated osteoclast cells after γ irradiation at a range of doses between 0.1 and 1 Gy in RAW 264.7 cells. Analysis of survival of the cells indicated a typical dose response, consistent with the reported study showing that these cells are radiosensitive [35]. The cell concentration after 1 Gy γ irradiation was about 60% of the non-irradiated samples after a 5-day culture. However, correcting the number of quantified GMCs for radiation-induced decreased cell growth did not changed the output significantly, suggesting that the results presented were not primarily due to the cell killing effects (Figure 1). The dose response for the formation of MNCs was interesting. Radiation alone apparently stimulated MNC formation at doses as low as 0.1 Gy, particularly for GMCs that are relevant to bone resorption. GMC formation peaked at doses around 0.5 Gy and decreased for higher doses (Figure 2). The morphological data are consistent with the gene expressions for *Trap*, *Ocstamp* and *Dcstamp*, as shown in Figure 4. It should be noted that the window of doses for enhancement of MNCs between 0.1–0.5 Gy is relevant to space radiation exposures. In RAW 264.7 cells, γ rays have also been shown to promote osteoclast function, but at a dose of 2 Gy [36].

While the formation of MNCs under simulated microgravity for RAW 264.7 cells have been reported previously [7,26], our study was the first to investigate the combined effects of microgravity and radiation for this cell type. The present study confirmed that simulated microgravity alone stimulated the formation of MNCs, but the level of MNCs depended apparently on the concentration of RANKL (Figure 1) and the duration of osteoclast culture [26]. Osteoclasts in the present study were quantified by GMCs containing 10 or more nuclei. At 50 ng/mL concentration of RANKL, simulated microgravity alone would double the number of GMCs over the background (Figure 1 and Figure 3). With combined exposure to radiation and microgravity, the number of GMCs increased as the dose of ionizing radiation increased, reaching a peak for doses around 0.1–0.5 Gy and decreased as the dose increased further beyond 0.5 Gy (Figure 3). Bone loss under partial weight bearing is another area of research interest [37,38] but is not addressed in the present study.

**Figure 5:**
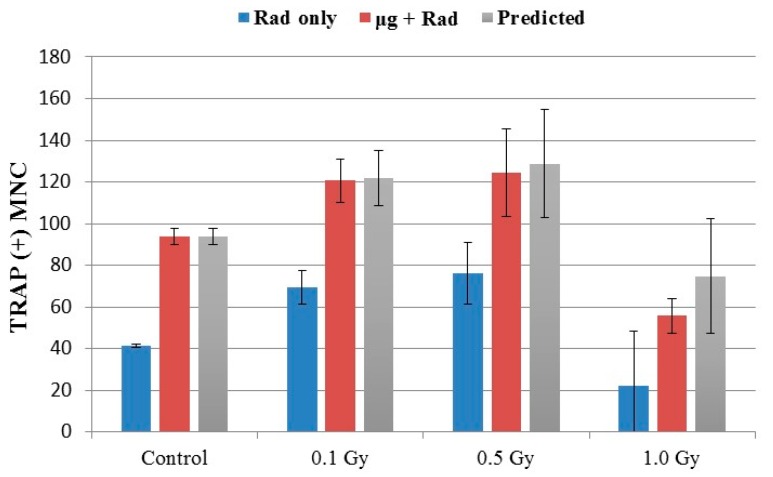
Osteoclast fusion prediction. Blue and red bars represent MNCs containing ≥10 nuclei analyzed in Figure 3 for radiation alone and combined radiation and microgravity exposures, respectively. Microgravity alone induced about 52 GMCs (=94 − 42) per dish in comparison to the background (0 Gy). The predicted number based on additive effects of radiation and microgravity (gray bars) is then the sum of this number and the number of GMCs after irradiation alone. For all three doses of 0.1, 0.5, and 1 Gy, the combined effects (red bars) agreed well with the prediction calculated based on the additive effects (gray bars).

One of the fundamental questions in space biology research is whether the combined biological effects of microgravity and exposure to cosmic radiation are synergistic. While studies addressing this question have been carried out for half a century in space or using simulated microgravity on the ground, the reported results have been conflicting, at least for DNA damage response endpoints [15,39]. With regard to bone loss, the combined effects have been reported in studies using mostly rodents with hindlimbs elevated to simulate the effects of skeletal unloading (HU) while being exposed to radiation [1,16,30,32,40,41]. Some of these studies reported the radio-sensitizing effects of hindlimb unloading in some, but not all bones. To determine possible synergism for MNC formation, we present, in Figure 5, the number of GMCs by radiation alone, and combined radiation and microgravity, against the predicted number based on the additive effects. It is shown that microgravity alone induced about 52(=94 − 42) GMCs per dish in comparison to the background (Figure 5, 0 Gy). The predicted number based on additive effects (gray bar in Figure 5) was then the sum of this number and the number of GMCs for irradiated cells. For all three doses of 0.1, 0.5, and 1 Gy, the GMC number in cells after combined exposure to microgravity and radiation agreed well with the prediction, suggesting that the effects of these two factors were additive. It is interesting to note that microgravity enhanced GMCs (~52 MNCs/dish) at levels that are more than that of radiation-enhanced (~33 MNCs/dish), even at the peak radiation dose of 0.1 or 0.5 Gy, suggesting that microgravity would contribute more to osteoclast differentiation than radiation in the presence of these two factors in space.

In the present study, we assessed the expression of key genes involved in osteoclasts differentiation. No significant dysregulation of the *Ctfg* gene was observed, either for radiation alone or combined with simulated microgravity (Figure 4). Expressions of *Dcstamp* and *Ocstamp* peaked for doses of 0.1–0.5 Gy, but the fold changes for radiation alone were significantly lower than changes caused by microgravity (Figure 4). Such differences were particularly pronounced for *Ocstamp*. Expressions of *Ocstamp* and *Dcstamp* under simulated microgravity alone have been reported previously [7,26,42]. However, the observation that the microgravity effect on *Ocstamp* and *Dcstamp* expression is higher than the radiation effect suggests that radiation might not increase the synthesis of adhesion molecules as much as microgravity, and this could explain the lower number of GMCs formed after radiation when compared to microgravity. However, the protein levels and functionality of *Ocstamp* and *Dcstamp* need to be addressed in order to enforce these findings. Furthermore, we also analyzed expressions of *Trap*. TRAP is expressed in osteoclasts and is able to degrade skeletal phosphoproteins including osteopontin (OPN) [43]; therefore, TRAP has been associated with bone resorption [44]. In the present study, TRAP expression was induced by radiation, microgravity, and the combination of both (Figure 4). However, in contrast to *Ocstamp* and *Dcstamp*, upregulation of TRAP was higher in irradiated cells than in cells exposed to microgravity. Thus, it might be the case that radiation predominantly affects bone resorption while microgravity affects the formation of GMCs.

## 4. Materials and Methods

### 4.1. Cell Culture in Simulated Microgravity and γ Irradiation

**Figure 6:**
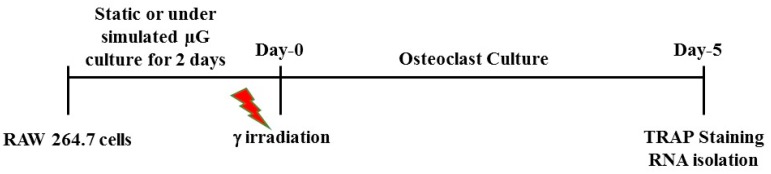
Timeline of the experiment. RAW 264.7 cells were cultured in static or under simulated microgravity for 48 h before exposed to varying doses of γ rays. Cells were then cultured in the presence of RANKL for 5 days for quantification of osteoclast differentiation and for gene expression analysis.

RAW 264.7 murine macrophage cells were purchased from American Type Culture Collection (Manassas, VA, USA) and maintained in static condition with Delbecco’s Modified Eagle’s medium (DMEM) with 10% fetal bovine serum (FBS) and 1% penicillin/streptomycin (Invitrogen, Carlsbad, CA, USA) in a humidified incubator at 37 °C with 5% CO_2_. NASA-developed ground-based rotating wall vessels (RWVs) were used to simulate the microgravity (µg) conditions. A Cs-137 γ source at NASA Johnson Space Center was used to deliver radiation of varying doses. Figure 6 shows the experimental timeline. RAW 264.7 cells suspended in complete media were cultured in rotation at 20 rpm or in a static condition for 48 h in a humidified incubator at 37 °C with 5% CO_2_. The cells were then removed from the incubator, and exposed to γ rays at doses of 0.1, 0.5, or 1 Gy. After irradiation, the cells were cultured for the formation of multinucleated osteoclasts. This study focused on doses of 1 Gy or lower that are relevant to space radiation exposure.

### 4.2. Cell Concentration and Viability

To determine viability of the cells after radiation exposure, irradiated and non-irradiated RAW 264.7 cells were seeded in the wells of 96-well plates at the density of 3 × 10^3^ cells/mL. Cells were then incubated in a 37 °C for 5 days in DMEM supplemented with 10% FBS and 1% L-glutamine. At the end of the 5-day culture period, the number of cells in a well was measured with a Coulter counter. Viability of irradiated cells with prior culture in simulated microgravity was also analyzed.

### 4.3. Osteoclast Culture

**Figure 7:**
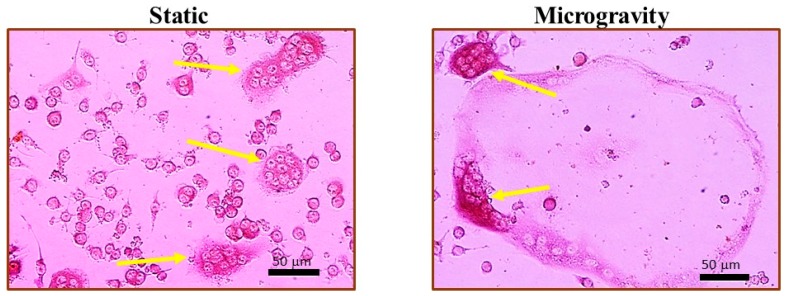
Multinucleated osteoclasts (pointed by arrows) formed in cells after a 5-day osteoclast culture in RANKL. Cells were maintained in static conditions or under simulated microgravity for 48 h immediately prior to osteoclast culture.

RAW 264.7 cells were cultured under static or stimulated microgravity conditions, and then exposed to different doses of γ rays. After exposure, cells previously cultured under different gravity conditions were further incubated under a static condition in the presence of RANKL (50 ng/mL) and M-CSF (10 ng/mL) (R & D Systems, Minneapolis, MN, USA) in order to stimulate osteoclast differentiation. After 5 days, cells were fixed and stained for tartrate resistant acid phosphatase (TRAP) activities using an Acid Phosphatase, Leukocyte (TRAP) Kit (Sigma, St Luis, MO, USA). TRAP positive multinucleated osteoclasts were scored with a Zeiss microscope. Figure 7 shows the examples of TRAP positive multinucleated cells (MNCs). To quantify osteoclast differentiation, MNCs were grouped by the number of nuclei in a cell. Giant multinucleated cells (GMCs) were classified by those containing 10 or more nuclei [45].

For the induction of MNCs, non-irradiated RAW 264.7 cells were incubated under static or rotating conditions with two different concentrations of RANKL: 25 ng/mL and 50 ng/mL.

### 4.4. Real-Time Polymerase Chain Reaction (RT-PCR) Analysis

At the end of 5 days of osteoclast culture, total RNA was isolated from cells previously cultured under the static or simulated microgravity conditions, with or without radiation exposure. Total RNA was reverse transcribed using random hexamers and Moloney murine leukemia virus reverse transcriptase (Invitrogen, Carlsbad, CA, USA). The resulting cDNAs were then subject to quantitative real-time reverse transcription polymerase chain reaction using specific primers for DCSTAMP, OCSTAMP, CCN2/CTGF, and TRAP as osteoclast markers. Relative levels of gene expressions were normalized in all the samples analyzed with respect to the levels of GAPDH amplification. Primers were designed and ordered from Invitrogen (Carlsbad, CA, USA), and the sequences were as follows: TRAP 5′-CGACCATTGTTAGCCACATACG-3′ (sense) and 5′-TCGTCCTGAAGATACTGCAGGTT-3′ (anti-sense); CCN2/CTGF 5′-CCACCCGAGTTACCAATGAC-3′ (sense) and 5′-GTGCAGCCAGAAAGCTCA-3′ (anti-sense); DCSTAMP 5′-CTAGCTGGCTGGACTTCATCC-3′ (sense) and 5′-TCATGCTGTCTAGGAGACCTC-3′ (anti-sense); GAPDH 5′-GCCAAAAGGGTCATCATCTC-3′ (sense) and 5′-GTCTTCTGGGTGGCAGTGAT-3′ (anti-sense); OCSTAMP 5′-GGCAGCCACGGAACAC-3′ (sense) and 5′-GCAGGGGGTCCCAAAG-3′ (anti-sense). RT-PCR analysis were performed at 94 °C for 4 min, followed by 35 cycles of amplification at 94 °C for 30 s, 58 °C for 1 min, 72 °C for 2 min, and 72 °C for 10 min as the final elongation step.

### 4.5. Statistical Analysis

The experiments were performed three times independently. In each of the experiments, cells were cultured in three RWV or dishes for each of the gravity and radiation dose conditions. Significant differences were analyzed by one-way ANOVA for comparing effects within one group and two-way ANOVA for comparing two groups. We used Prism version 6 as statistical software (GraphPad Software, La Jolla, CA, USA). For analyzing the effect of radiation on osteoclast fusion (Figure 3), a *t*-test was applied comparing each dose with 0 Gy.
